# Esophageal carcinoma with ductal differentiation combined with multiple myeloma: a case report

**DOI:** 10.3389/fonc.2026.1922758

**Published:** 2026-09-30

**Authors:** Hongjing Sun, Minghao Gao, Xiaohui Feng

**Affiliations:** 1 Department of Pathology, Hebei Medical University Third Hospital, Shijiazhuang, China; 2 School of Basic Medicine, Hebei Medical University, Shijiazhuang, China

**Keywords:** endoscopic submucosal dissection, esophageal carcinoma with ductal differentiation, immunohistochemistry, multiple myeloma, synchronous multiple primary malignancies

## Abstract

Esophageal carcinoma with ductal differentiation (ECDD) is a rare esophageal cancer (EC), characterized by distinctive morphological features, including bilayered duct-like structures and a biphenotypic immunophenotype. Multiple myeloma (MM) is a plasma cell neoplasm. The synchronous occurrence of these two entities is exceedingly rare. We report an 80-year-old male who presented with fatigue, anemia, and hypoalbuminemia. Endoscopic examination revealed a high-grade intraepithelial neoplasia of the esophagus. Following endoscopic submucosal dissection, pathological examination confirmed ECDD. Immunohistochemistry demonstrated that the inner layer cells of the duct-like structures were positive for CK7 and CAM5.2, while the outer layer cells were positive for p40 and p63. Concurrently, elevated serum globulin levels prompted further evaluation, leading to a diagnosis of MM. At 13 months postoperatively, no recurrence or metastasis of EC was observed, and the patient’s MM remained stable. This case highlights that in elderly patients presenting with hematological abnormalities, the possibility of coexisting solid malignancy and plasma cell dyscrasia should be considered. ECDD has distinctive pathological features, and accurate recognition is critical to avoid misdiagnosis.

## Introduction

Esophageal cancer (EC) ranks among the most common malignancies worldwide, with squamous cell carcinoma and adenocarcinoma being the predominant histological types (1). Esophageal carcinoma with ductal differentiation (ECDD) is a rare EC that has been increasingly documented in recent literature. It is morphologically characterized by distinctive bilayered duct-like structures, consisting of an inner luminal epithelial layer and an outer basal epithelial layer, with a biphenotypic immunoprofile (2–4).

Multiple myeloma (MM) is a clonal plasma cell proliferative disorder that may be accompanied by second primary malignancy (SPM). Published data indicate that 2.75% of patients with MM had a preceding primary malignancy before the diagnosis of MM (5). The synchronous occurrence of EC and MM is extremely unusual, and to date, no case of ECDD combined with MM has been reported in the literature. Herein, we describe an elderly patient with concurrent ECDD and MM, and discuss the clinicopathological features and therapeutic considerations.

## Case presentation

### 1 Admission history

An 80-year-old male was found to have hypoalbuminemia and anemia on a routine health examination two months prior, accompanied by persistent fatigue of unknown origin. Two weeks before admission, further workup at a local hospital revealed moderate anemia, elevated globulin, and an inverted albumin-to-globulin ratio. Esophagogastroduodenoscopy identified an esophageal mucosal lesion, and biopsy pathology reported “high-grade intraepithelial neoplasia”. The patient was referred to our institution for further management.

Since symptom onset, the patient’s general condition remained fair, with no significant dysphagia, retrosternal pain, or other gastrointestinal complaints. His medical history included hypertension for over 20 years and benign prostatic hyperplasia for 3 years. He denied any history of smoking, alcohol consumption, or family history of malignancy.

### 2 Diagnostic evaluation on admission

On repeat esophagogastroduodenoscopy (**Figure 1**), a reddish, coarse mucosal area was observed at 30–35 cm from the incisors, with multiple whitish granular protrusions. Magnifying endoscopy with narrow-band imaging demonstrated vascular patterns of type B1–B2, with focal suspicion of type B3. Lugol’s chromoendoscopy showed an unstained area. According to the Japan Esophageal Society AB classification (6), type B1 suggests invasion to the epithelial layer or lamina propria mucosa (M1–M2), type B2 suggests invasion to the muscularis mucosa or submucosa ≤ 200 μm (M3–SM1), and type B3 suggests submucosal invasion > 200 μm (SM2 or deeper). Endoscopic ultrasonography with a miniature probe (**Figure 1**) demonstrated thickening of the mucosa and submucosa at the lesion, measuring approximately 4.2 mm, with an intact and continuous muscularis propria. Chest CT revealed possible focal wall thickening in the lower esophagus.

**Figure 1:**
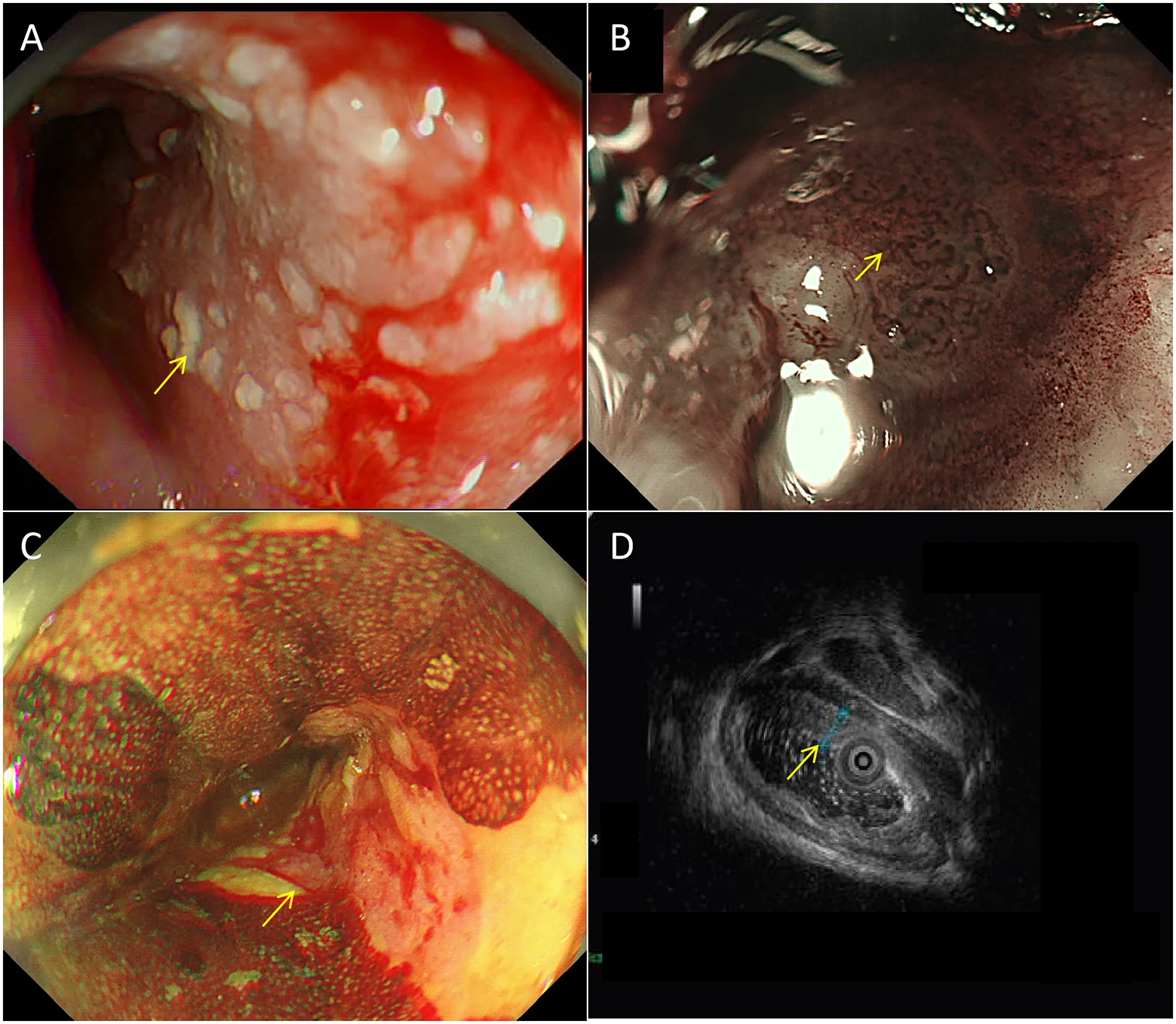
**(A)** The lesion showed reddish, coarse mucosa with multiple whitish granular protrusions (yellow arrow). **(B)** Magnifying endoscopy with narrow-band imaging revealed vascular patterns of type B1–B2, with focal suspicion of type B3 (yellow arrow). **(C)** Lugol’s chromoendoscopy demonstrated an unstained area (yellow arrow). **(D)** Endoscopic ultrasonography with a miniature probe demonstrated thickening of the mucosa and submucosa at the lesion, approximately 4.2 mm, with an intact and continuous muscularis propria (yellow arrow).

### 3 Endoscopic submucosal dissection and postoperative pathology

Given the patient’s advanced age, poor physical tolerance, and concurrent hematologic abnormalities, the risk of radical esophagectomy was deemed prohibitively high. After full informed consent was obtained from the patient and his family, endoscopic submucosal dissection (ESD) was elected. This procedure served the dual purpose of providing definitive pathological staging and achieving local therapeutic control.

After preoperative preparation, ESD of the esophageal mucosal lesion was performed under general anesthesia with endotracheal intubation. Intraoperatively, a Dual knife was used to mark the periphery approximately 0.5 cm outside the lesion margin. After submucosal injection, the lifting sign was positive. The incision was made along the marked points, and the lesion was completely dissected and submitted for pathological examination.

Pathological findings:

Gross findings: A piece of mucosal tissue measuring 4.7 × 3.5 × 0.3 cm was received, with a rough area of 2 × 1.5 cm on the mucosal surface. The cut surface was gray-white and firm.

Microscopic findings (**Figure 2**): The tumor predominantly exhibited distinctive bilayered duct-like structures, composed of an inner luminal epithelial layer and an outer basal epithelial layer. The overlying squamous epithelium showed high-grade intraepithelial neoplasia. A morphological continuity is observed between the high-grade squamous intraepithelial neoplasia and the invasive duct-forming carcinomatous component. No Barrett’s esophagus or ectopic gastric mucosa was identified in the background esophageal mucosa.

**Figure 2:**
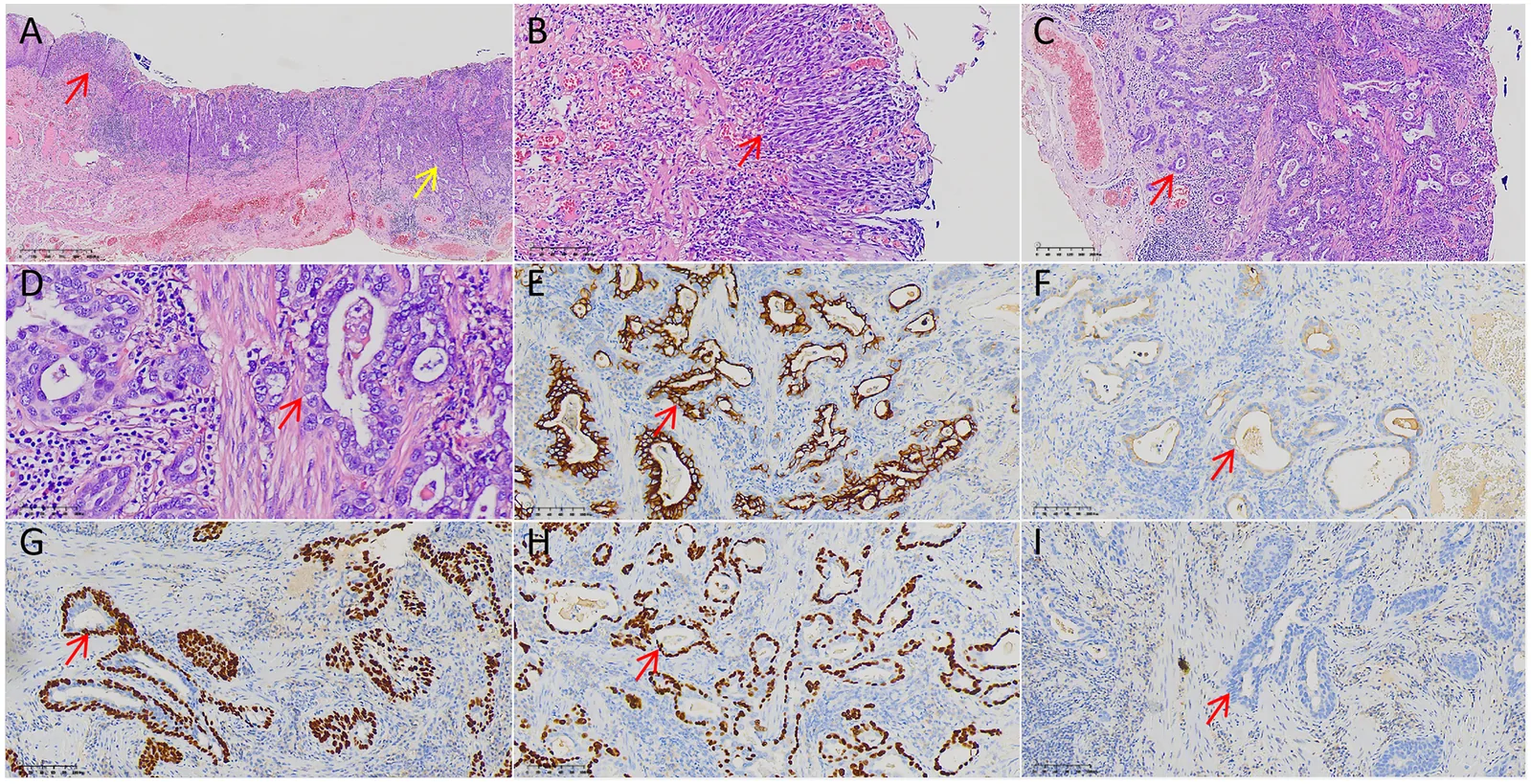
**(A)** A morphological continuity is observed between the high-grade squamous intraepithelial neoplasia and the invasive duct-forming carcinomatous component (HE, ×40; red arrow: high-grade intraepithelial neoplasia, yellow arrow: invasive duct-forming carcinomatous component). **(B)** Overlying squamous epithelium showing high-grade intraepithelial neoplasia (HE, ×200; red arrow). **(C)** Tumor tissue with bilayered duct-like structures infiltrating into the submucosa (HE, ×100; red arrow). **(D)** Distinctive bilayered duct-like structure with an inner luminal epithelial layer and an outer basal epithelial layer (HE, ×400; red arrow). **(E)** Inner glandular epithelial cells positive for CK7 (IHC, ×200; red arrow). **(F)** Inner glandular epithelial cells weakly positive for CAM5.2 (IHC, ×200; red arrow). **(G)** Outer basal epithelial cells positive for p63 (IHC, ×200; red arrow). **(H)** Outer basal epithelial cells positive for p40 (IHC, ×200; red arrow). **(I)** Bilayered duct-like structures showing p53 with a completely absent (null-type) aberrant expression pattern (IHC, ×200; red arrow).

Immunohistochemistry (IHC) (**Figure 2**): Inner layer glandular epithelium: CK7 (+), CAM5.2 (weak +); outer layer basal epithelium: p40 (+), p63 (+); p53 (null-type); Ki-67 (20%+); Desmin (smooth muscle +); S-100(-); CD117(-); CDX2 (-); Villin (-); SYN (-); mismatch repair proteins were all positively expressed.

Pathological diagnosis: Esophageal carcinoma with ductal differentiation (stage pT1b-SM2, with submucosal invasion to a depth of 700 μm), with no evidence of nerve or vascular invasion. High-grade intraepithelial neoplasia of squamous epithelium was present in the surrounding mucosa. Resection margins were negative for carcinoma and intraepithelial neoplasia.

### 4 Detection of comorbidity and diagnosis of multiple myeloma

Post-admission laboratory findings: Hemoglobin 65 g/L, serum calcium 2.56 mmol/L, creatinine 104 μmol/L. 24-hour urine protein quantification 0.286 g/d. Marked hyperglobulinemia (77.7 g/L) with concomitant hypoalbuminemia (30.8 g/L), and significantly elevated erythrocyte sedimentation rate (ESR, 152 mm/h). The above findings were suggestive of possible concurrent autoimmune disease or plasma cell dyscrasia. Antinuclear antibody panel was negative. Serum protein electrophoresis and immunofixation electrophoresis revealed: IgA 33.400 g/L, lambda light chain 7.190 g/L, serum free kappa light chain 9.87 mg/L, serum free lambda light chain 397.98 mg/L, with a free kappa/lambda light chain ratio of 0.0248. A high suspicion of MM was raised, and a hematology consultation was requested. Bone marrow aspiration and relevant ancillary studies were subsequently performed from the right posterior superior iliac spine. The results were as follows:

Urine immunofixation electrophoresis: Positive for monoclonal free lambda light chains, with a quantitative level of 15.7 mg/dL.

Flow cytometry: Abnormal plasma cells accounted for 23.71% of nucleated cells, expressing CD38, CD138, CD56, CD28, and cLambda, with weak expression of CD27 and CD81, and negative for CD45, CD19, CD20, CD117, and cKappa.

Bone marrow smear (**Figure 3**): Immature plasma cells comprised 42%, and mature plasma cells 4%, consistent with a myelomatous bone marrow pattern.

**Figure 3:**
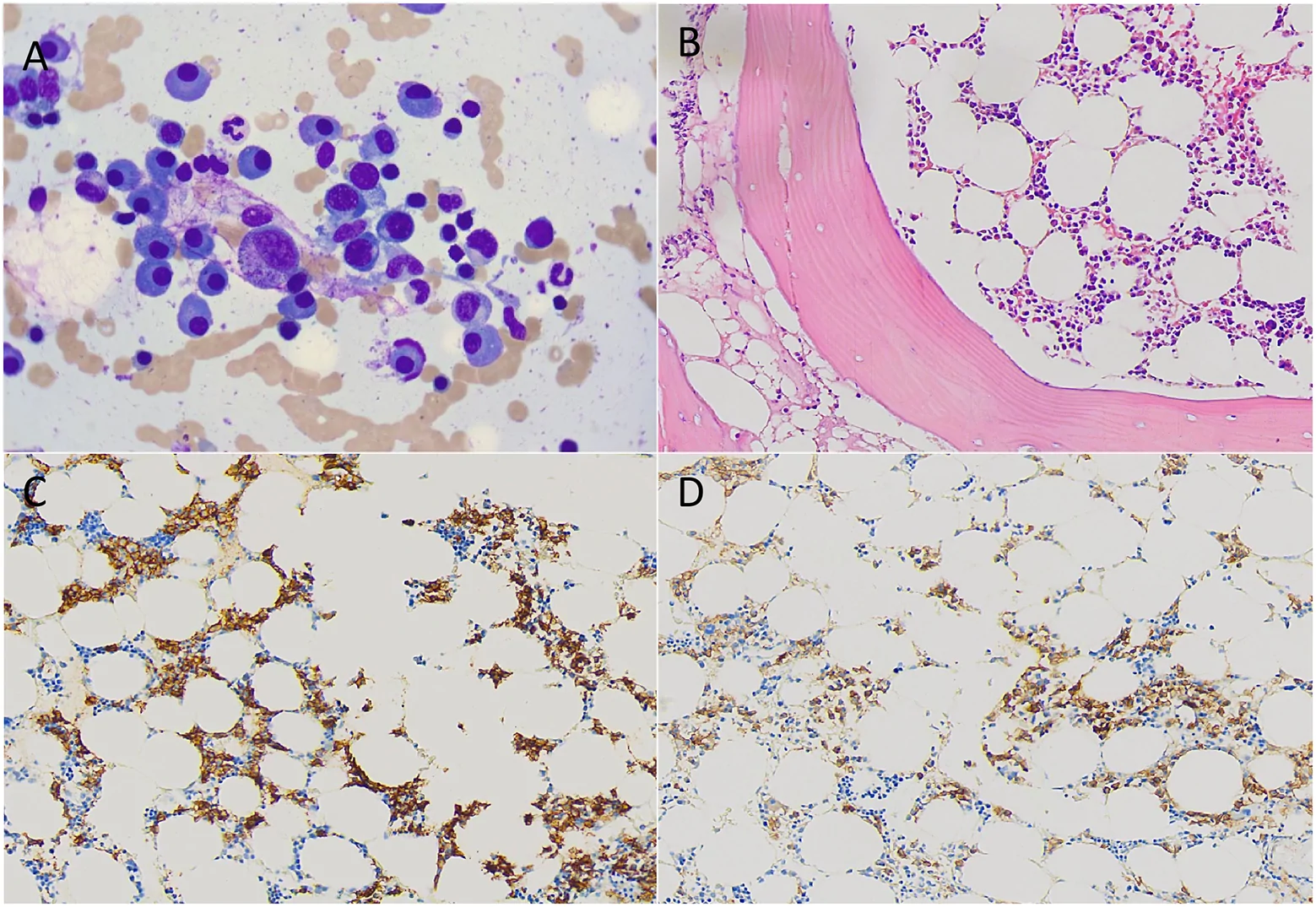
**(A)** Increased proportion of abnormal plasma cells (bone marrow smear). **(B)** Numerous abnormal plasma cells scattered or clustered, with abundant cytoplasm and eccentric nuclei (HE, ×200). **(C)** Abnormal plasma cells positive for CD138 (IHC, ×200). **(D)** Abnormal plasma cells positive for CD38 (IHC, ×200).

Bone marrow biopsy (**Figure 3**): Numerous abnormal plasma cells were identified, distributed either scattered or in clusters (approximately 20% of cellularity), with abundant cytoplasm and eccentric nuclei. IHC showed CD38 (+), CD138 (+), CD56 (+),CD34 (-), CD117 (-), MPO (-), E-Cadherin (-), CD61 (-), and CD20 (-); light chains was rendered uninterpretable (kappa negative and lambda negative) due to the effects of decalcification; therefore, clonality assessment was determined based on flow cytometry results; consistent with a plasma cell neoplasm.

Bone marrow FISH: Detected t(4;14) translocation and CKS1B (1q21) amplification.

Radiographic findings: Whole-spine MRI revealed abnormal bone marrow signal intensity involving the vertebral bodies and posterior elements of the cervical, thoracic, and lumbar spine, as well as portions of the sacrum. No well-defined, measurable focal lytic lesions were identified. On T1-weighted images, multiple scattered punctate hypo- to isointense foci were noted within the spinal bone marrow. T2-weighted images showed no overt abnormal signal intensity, although the marrow signal was overall heterogeneous. On fat-suppressed sequences, the spinal bone marrow demonstrated a diffuse, relatively homogeneous mildly hyperintense signal, without isolated or multiple, dimensionally measurable focal hyperintense nodules or masses.

Overall diagnosis: High-risk multiple myeloma [t(4;14) translocation and CKS1B (1q21) amplification] (7).

### 5 Treatment and follow-up

Post-ESD pathology indicated a depth of invasion of 700 μm (SM2), exceeding the absolute indication for curative ESD. Although the resection margins were negative, additional therapy was indicated according to current guidelines (8). However, given the patient’s advanced age and coexisting high-risk MM, the risk of radical esophagectomy was prohibitively high, and radiotherapy might have interfered with the systemic treatment course for MM. Following multidisciplinary team (MDT) discussion and thorough communication with the patient and family members, it was decided to defer additional therapy for esophageal cancer and prioritize the initiation of systemic therapy for MM. Postoperatively, given the extensive wound surface (4.7 cm × 3.5 cm), prednisone acetate was administered to prevent esophageal stricture. MM was treated with the BRD regimen (bortezomib, lenalidomide, dexamethasone).

Follow-up: Gastroscopy at 4 mo postop: esophageal stricture, no local recurrence. Regular chest CT q3mo. At last follow-up (13 mo postop): no local recurrence or lymph node metastasis, and the MM remained stable under regular BRD regimen chemotherapy. For a clear overview of the full diagnostic and treatment course from presentation to last follow-up, see the table below for a timeline of key clinical events (**Table 1**).

**Table 1:** Diagnostic and treatment timeline.

Time point	Key clinical events
2 months prior to admission	Physical examination revealed hypoalbuminemia and anemia, accompanied by unexplained persistent fatigue.
2 weeks prior to admission	Gastroscopy at a local hospital revealed an esophageal mucosal lesion; biopsy pathology diagnosed as “high-grade intraepithelial neoplasia.”
Current admission	Upon admission, gastroscopy, endoscopic ultrasound and CT were repeated to evaluate the esophageal lesion. Laboratory findings revealed markedly elevated globulin levels, raising suspicion of a plasma cell disorder.
Post-admission (ESD)	ESD was performed with complete en bloc resection of the esophageal lesion. Postoperative pathology confirmed ECDD (pT1b-SM2 stage).
Concurrent MM diagnosis	In parallel, due to the abnormal globulin elevation, bone marrow aspiration, immunofixation electrophoresis, FISH, and ancillary studies were completed, culminating in a definitive diagnosis of high-risk MM.
Initial post-treatment	Prednisone acetate was administered to prevent esophageal stricture. Following multidisciplinary discussion, adjuvant therapy for esophageal cancer was deferred, and systemic therapy for MM was prioritized.
4 months postoperatively	Follow-up gastroscopy revealed esophageal stricture formation, with no evidence of local tumor recurrence.
13 months postoperatively (last follow-up)	Regular surveillance (chest CT every 3 months) showed no recurrence or metastasis of esophageal cancer; MM remained stable after chemotherapy with the BRD regimen.

## Discussion

### 1 Clinicopathological features and disease taxonomy of esophageal carcinoma with ductal differentiation

ECDD is a rare malignancy of the esophagus, predominantly affecting elderly males and commonly located in the middle to lower thoracic esophagus (2–4, 9–14). The present case is consistent with the age, sex, and anatomical location reported in the literature. Histologically, the hallmark is a bilayered duct-like structure: an inner luminal layer expressing glandular markers such as CK7, CK18, and CAM5.2, and an outer basal layer expressing squamous markers including p40, p63, and CK5/6 (3, 4, 10). This distinctive bilayer configuration serves as the core criterion for recognizing this morphological subtype. In the present case, typical bilayered duct-like structures were also observed, with the inner cells positive for CK7 and weakly positive for CAM5.2, and the outer cells positive for p40 and p63. Mismatch repair proteins showed intact expression, indicating proficient mismatch repair function, consistent with previous reports (9). Furthermore, Jiang et al. (4) classified 17 cases into two patterns: 52.9% as predominantly ductal differentiation carcinoma (squamous component <10%), and 47.1% as predominantly other carcinoma types (ductal differentiation component <10%). The present case falls into the former category.

Regarding ECDD taxonomy: the ECDD described here is currently used as a descriptive morphological term for esophageal carcinoma with bilayered duct-like structures and biphenotypic markers. ECDD has not yet been incorporated into the current WHO classification of esophageal tumors (15), and its nosological status warrants further clarification. The nosological borders between this tumor type and conventional esophageal SCC, carcinomas with esophageal gland/submucosal gland differentiation, and other salivary gland-like esophageal carcinomas require further case accumulation and molecular pathology studies. Thus, ECDD is used here as a descriptive morphological diagnosis, not as a recognized independent subtype in the current classification.

Aberrant p53 protein expression is one of the common features of this tumor category; however, it is not specific to ECDD and should be interpreted in conjunction with morphological findings for comprehensive assessment. It has been reported that the TP53 mutation rate is extremely high in esophageal squamous cell carcinoma, with a 94.44% concordance between mutant-type p53 expression and TP53 gene mutations, achieving 100% diagnostic sensitivity and specificity. Mutant-type p53 expression can serve as a surrogate marker for TP53 mutations detected by WES or WGS and aids in the differentiation of benign from malignant esophageal lesions (16). In ECDD, the TP53 mutation rate is also high (10). Du et al. (3) reported 8 cases, of which 6 showed diffuse strong positivity and 2 showed loss of expression. Zhu et al. (10) reported 9 cases, all showing diffuse strong positivity. This case shows p53 (null-type), indicating possible TP53 loss-of-function alteration. However, IHC for p53 has limitations and cannot replace sequencing for defining the exact mutation type.

Regarding histogenesis, the bilayered duct-like morphology of this tumor suggests differentiation toward the ducts of the esophageal submucosal glands, but the direction of differentiation does not necessarily equate to the tissue of origin. Some authors have proposed a possible origin from the submucosal ducts, whereas others contend that it merely represents ductal glandular differentiation (12, 13). In the present case, a morphological continuity is observed between the high-grade squamous intraepithelial neoplasia and invasive duct-forming carcinomatous component suggests that they may be of common origin. However, morphological transition per se cannot distinguish between biphenotypic differentiation within the same clone and a collision tumor arising from distinct clones; therefore, it is inappropriate to extrapolate the exact tissue of origin based solely on this observation. Notably, none of the reported cases have shown columnar metaplasia, intestinal metaplasia, or ectopic gastric mucosa in the overlying squamous epithelium, suggesting that this disease does not arise from Barrett’s esophagus or ectopic gastric mucosa (3, 4). The overlying squamous epithelium in this case also showed no such changes, supporting this view. The current case also supports this view.

Differential Diagnoses: 1. Esophageal ductal adenoma: bilayered but non-invasive, without atypia, and with wild-type p53 expression (3); 2. Adenosquamous carcinoma: independent squamous and glandular components without bilayered ductal structures (3); 3. Adenoid cystic carcinoma: usually submucosal, with normal overlying mucosa, composed of ductal and myoepithelial cells in cribriform, tubular, or solid patterns, with myoepithelial markers S-100 and CD117 positivity and the presence of MYB/NFIB fusion gene (10, 11); In the present case, S-100 and CD117 were negative, and the histomorphological findings did not support this diagnosis; if clinically highly suspected, MYB-related molecular testing may be performed for further differentiation. 4. Mucoepidermoid carcinoma: composed of epidermoid, intermediate, and mucous cells, with MAML2 rearrangement (10, 11). The morphological and immunophenotypic features of the present case did not conform to the aforementioned characteristics. Furthermore, the present case was negative for CDX2 and Villin, which, in conjunction with the histomorphological and immunophenotypic findings, excluded intestinal-type adenocarcinoma. Based on the characteristic bilayered architecture and immunophenotype, the diagnosis of ECDD was confirmed.

### 2 Indications and therapeutic selection for endoscopic submucosal dissection

ESD indications: Stages M1 and M2 are absolute indications; stages M3 and SM1 are relative indications; for stages SM2 and SM3, the lymph node metastasis rate reaches 19%–56%, and ESD is not recommended (8, 17). In our case, the invasion depth of 700 μm (SM2) exceeded the indication, according to guideline standards, ESD is no longer considered curative treatment, and additional postoperative therapy should be administered (8). However, the patient was 80 years old with coexisting high-risk MM, and radical surgery/radiotherapy posed excessively high risks while also delaying MM treatment; conversely, deferring therapy carried the risk of lymph node metastasis. After full disclosure of the bidirectional risks, the patient and family members opted to prioritize MM control and defer additional therapy for EC. It should be clearly understood that ESD in this case served the dual purpose of accurate pathological staging and local tumor debulking, and the decision not to administer postoperative additional therapy was arisk-benefit-balanced decision based on the patient’s individual circumstances, rather than implying that ESD alone is a recommended curative modality for SM2-stage esophageal cancer. Currently, there is no high-level evidence-based medical evidence for the concurrent treatment of ECDD and MM. At the 13-month postoperative follow-up, gastroscopy and chest CT revealed no local recurrence or lymph node metastasis; however, the follow-up period is relatively short and insufficient to confirm the long-term oncological safety of this strategy, and the long-term efficacy requires further observation.

The incidence of stricture after extensive esophageal ESD is extremely high. For lesions with a longitudinal resection length of less than 5 cm, the incidence of refractory stricture is 34%; for lesions with a length of 5 cm or greater, the incidence reaches 83% (18). In the present case, the wound size was 4.7 × 3.5 cm, indicating a relatively high risk. Studies have shown that oral prednisone combined with a proton pump inhibitor can effectively prevent esophageal stricture (19). Another study demonstrated that oral prednisone alone or oral prednisone combined with local triamcinolone injection can effectively prevent esophageal stricture, reduce the frequency of endoscopic dilation, and prolong the time interval to the first dilation after ESD (20). In this case, the post-ESD mucosal defect measured 4.7 × 3.5 cm, representing a high-risk condition for stricture formation. Despite prophylactic administration of prednisone acetate, esophageal stricture still developed, indicating that stenosis may occur even with preventive medication in patients undergoing extensive resection. However, patient currently has adequate oral intake, so no intervention given. If oral intake severely affected, endoscopic balloon dilation should be considered.

### 3 Concurrent multiple myeloma and esophageal carcinoma with ductal differentiation

MM may be accompanied by SPM. The literature reports that 6.07% of patients with MM may develop SPM, and 2.75% of patients had a primary malignancy prior to the diagnosis of MM, with prostate cancer being the most common solid tumor (5). The occurrence of SPM in MM is multifactorial and complex, which may be associated with disease-related risk factors, host-related risk factors, and treatment-related risk factors (21). However, in the present case, the patient had a history of anemia and esophageal lesions prior to the diagnosis of MM, and the diagnoses of ECDD and MM were made almost synchronously. Therefore, they should be accurately characterized as synchronous multiple primary malignancies, rather than ECDD being a secondary SPM arising from MM. Multiple primary malignancies (MPMs) are defined as two or more histologically confirmed primary malignancies occurring in the same patient, which may arise from the same or different organs or different systems, and do not include metastases from the primary cancer (22). The most common presentation of MPMs is double primary malignancies, which can be classified into two types according to the time interval between diagnoses. Synchronous malignancies are defined as the second tumor occurring simultaneously with or within 6 months of the diagnosis of the first malignancy, whereas metachronous malignancies are defined as the second tumor occurring 6 months or more after the diagnosis of the first malignancy (23). In the present case, the patient had a history of anemia and esophageal lesions before the diagnosis of MM. Therefore, MM and ECDD were most likely synchronous double primary malignancies rather than therapy-related second neoplasms. In the present case, the two tumors were diagnosed at nearly the same time, consistent with synchronous double primary malignancies. However, a single synchronous case is insufficient to establish a biological association between the two entities. Furthermore, the MM in this case harbored high-risk cytogenetic abnormalities, including t(4;14) translocation and CKS1B (1q21) amplification, as detected by FISH (7). Currently, there is no evidence to suggest a causal relationship between these genetic alterations and the development of ECDD. Their coexistence more likely reflects an incidental clustering of tumorigenesis in the elderly population.

## Conclusion

We report a rare case of ECDD coexisting synchronously with MM in an 80-year-old male patient. ECDD is characterized by distinctive bilayered duct-like structures and immunophenotypic morphological features. In elderly patients presenting with hematological abnormalities, the possibility of coexisting solid tumors and plasma cell disorders should be considered. A multidisciplinary team approach is essential for accurate diagnosis and individualized treatment of such complex cases. The short-term follow-up (13 months) in this case is not yet sufficient to adequately support the long-term safety of the treatment strategy, and a longer period of observation is required.

## Data Availability

The original contributions presented in the study are included in the article/supplementary material. Further inquiries can be directed to the corresponding author.
